# Supplementary White, UV-A, and Far-Red Radiation Differentially Regulates Growth and Nutritional Qualities of Greenhouse Lettuce

**DOI:** 10.3390/plants12183234

**Published:** 2023-09-12

**Authors:** Zhengnan Yan, Chunling Wang, Zhixin Li, Xin Li, Fei Cheng, Duo Lin, Yanjie Yang

**Affiliations:** 1 College of Horticulture, Qingdao Agricultural University, Qingdao 266109, China; yanzn@qau.edu.cn (Z.Y.); 20190202987@stu.qau.edu.cn (Z.L.);; 2 College of Water Resources and Architecture Engineering, Tarim University, Aral 843300, China; 120160023@taru.edu.cn; 3 College of Water Resources and Civil Engineering, China Agricultural University, Beijing 100083, China

**Keywords:** chlorophyll content, far-red light, principal component analysis, nutrient content, supplementary light

## Abstract

Light is a crucial environmental signal and a form of photosynthetic energy for plant growth, development, and nutrient formation. To explore the effects of light quality on the growth and nutritional qualities of greenhouse-grown lettuce (*Lactuca sativa* L.), lettuce was cultivated under supplementary white (W) light-emitting diodes (LEDs); white plus ultraviolet A LEDs (W+UV); white plus far-red LEDs (W+FR); and the combination of white, far-red, and UV-A LEDs (W+FR+UV) for 25 days, with lettuce grown under natural sunlight used as the control. The results indicate that the leaf length and leaf width values for lettuce grown under the W+FR+UV treatment were significantly higher than those of lettuce grown under other supplementary light treatments. The highest values of shoot fresh weight, shoot dry weight, root fresh weight, and root dry weight were recorded under the W+FR treatment (4.0, 6.0, 8.0, and 12.4 times higher than those under the control treatment, respectively). Lettuce grown under the W+FR treatment exhibited the highest total chlorophyll content (39.1%, 24.6%, and 16.2% higher than that under the W, W+UV, and W+FR+UV treatments, respectively). The carotenoid content of lettuce grown under the W+FR treatment was the highest among all treatments. However, the root activity of greenhouse-grown lettuce was the highest under the W+FR+UV treatment. Soluble sugar content, cellulose content, and starch content in the lettuce responded differently to the light treatments and were highest under the W+UV treatment. In summary, supplementary light promoted growth and nutrient accumulation in lettuce. Specifically, white plus far-red light promoted lettuce growth, and white plus UV increased some specific compounds in greenhouse-grown lettuce. Our findings provide valuable references for the application of light-supplementation strategies to greenhouse lettuce production.

## 1. Introduction

Lettuce (*Lactuca sativa*) has considerable market demand due to its high nutritional value and rapid growth rate. Therefore, the greenhouse cultivation area for lettuce has been gradually expanded to meet the growing needs of consumers in recent years [1]. Light is an important environmental factor in greenhouse vegetable production, with extensive regulatory effects on plant morphology, photosynthetic characteristics, physiological metabolism, and nutritional quality [2,3]. However, greenhouse light conditions, including light intensity, light quality, and photoperiod vary with seasons, latitudes, and the shielding by covering materials, structural framework, and equipment [4,5]. Supplementary artificial light is essential for vegetable production when greenhouse light conditions do not meet the requirements of plant growth [6]. Light-emitting diode (LED) fixtures are usually composed of LED lamps with different light wavelengths, which have been widely applied in horticultural production due to their monochromaticity, high photosynthetic radiation, and high electrical efficiency [7].

The use of light quality to regulate plant growth and morphology is an important technology for vegetable cultivation in the field of protected horticulture [8,9]. Plant pigments absorb light mainly within the spectral range of 400–700 nm, which is typically referred to as photosynthetic photon flux density (PPFD) [10]. Thus, numerous studies have investigated the manipulation of the PPFD and its impacts on the growth and phytochemical accumulation of lettuce [11,12,13]. Due to their broad-spectrum features, which are beneficial to plant growth, white (W) LEDs are commonly used for leafy-vegetable production [14,15]. However, the light spectrum of W-LEDs may not meet the production requirements of all types of plant species. For example, most W-LEDs lack short wavelengths, such as ultraviolet A (UV-A), which are important for plant growth and metabolism [16]. Therefore, adding other light to white light is beneficial for horticultural plants cultivated under artificial light. Plants use multiple photoreceptors to detect the properties of light in a wide waveband. For instance, cryptochrome is sensitive to blue and UV-A radiation [17], and phytochromes are particularly sensitive to red and far-red (FR) light [18]. The effects of ultraviolet light and far-red light on plant growth in horticultural vegetable production have gradually attracted widespread attention [19,20].

UV-A radiation has obvious correlations with plant morphology, physiology, biochemistry, and photosynthesis, but few scientific studies have been conducted to elucidate the signaling mechanisms governing such responses [21]. For example, the activity of phenylalanine ammonia-lyase (PAL) in lettuce and tomato has been widely reported to be increased by UV-A [22,23,24]; however, these studies only focused on PAL content change, and the related cause and complex mechanisms by which UV increases PAL remain to be further explored [24]. Some studies on the effects of UV-A on plant biomass obtained inconsistent results. One study found that adding UV-A to W-LEDs throughout the production period resulted in reduced lettuce biomass [25], while other studies reported that short-term preharvest lighting treatments with UV-A/blue (containing both UV-A and blue wavelengths) LEDs had no effect on lettuce growth and biomass [26,27]. Another study indicated that supplementing visible light with particular amounts of UV-A promoted tomato biomass accumulation [28]. UV-A has been reported to play a similar role to that of blue light in maintaining leaf photosynthetic function [29]. UV-A radiation notably affects secondary metabolism, and it is beneficial for the accumulation of many health-promoting phytochemicals and nutrients [30].

Far-red (FR) radiation is outside the PPFD waveband, and it can drive photochemistry and photosynthesis, although it has a low quantum yield [31]. Plant morphology (stem elongation and leaf expansion), seed germination, and flowering can be regulated by changing red and FR radiation, and phytochrome-mediated responses to these light changes in the environment have been extensively studied [32]. The addition of far-red radiation to red light can increase gibberellin content, accelerate cell division and cell elongation in the stem, and ultimately, increase plant height [33]. Although it is not a photosynthetic energy source, FR radiation increases the biomass of lettuce and tomato plants when it is combined with red LEDs [34,35]. Supplementation with FR light allows shade tolerance responses in some lettuce varieties, such as increases in leaf expansion and promotion of seedling growth to capture more light to drive photosynthesis [36,37].

Adding FR to red–blue light increases the leaf area and biomass production of lettuce in greenhouses [38]. One previous study indicated that the inclusion of FR light in W-LEDs does not affect the levels of most phytochemicals in lettuce, whereas the addition of FR light to UVA reduces the phytochemical content in lettuce [25]. Microgreens (basil, cabbage, kale, and kohlrabi) have been reported to be sensitive to the addition of FR light alone to W-LEDs, less sensitive to the addition of UV-A alone to W-LEDs, and insensitive to the addition of FR and UV-A to W-LEDs; thus, the combined addition of FR and UV-A to W-LEDs has no effects on growth and quality traits in microgreen plants [27]. Few studies have been conducted to reveal the effects of the combination of white, FR, and UVA radiation on the leaf morphology, growth, and nutritional quality of greenhouse-grown lettuce.

Hence, the objectives of this study were to investigate the effects of UV-A and/or FR radiation in addition to white light on the growth quality, biomass, and nutritional quality of lettuce. The effects of interactions between UV-A and FR radiation on the growth and nutrition of lettuce were also explored. Our findings support the establishment of guidelines for the application of light supplementation strategies in greenhouse lettuce production.

## 2. Results

### 2.1. Effects of Supplementary Light on Leaf Morphology of Greenhouse-Grown Lettuce

Different combinations of white, UV-A, and far-red light had significant effects on the leaf morphology of lettuce (Table 1). The result showed that the leaf length of lettuce treated with W+UV+FR was not significantly different from that of the control, but it was 21.1% higher than that of the W+UV group. The leaf length of lettuce grown under the W treatment was not significantly different from that grown under the W+FR treatment, but it was significantly higher than that grown under the W+UV treatment. All four supplemental light treatments resulted in a significantly larger leaf width than the control, and the leaf width of lettuce grown under the W+FR and W+FR+UV treatments was significantly larger than that grown under the W treatment.

### 2.2. Impacts of Supplemental Light on Pigment Content of Greenhouse-Grown Lettuce

**Figure 1:**
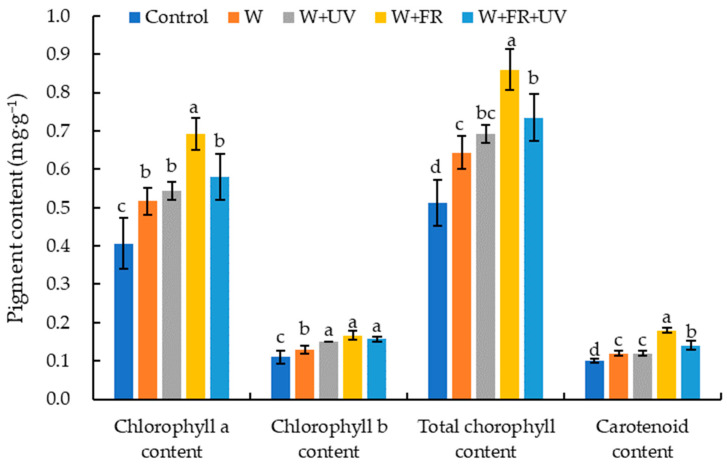
Pigment contents of greenhouse-grown lettuce under five treatments. W, white light-emitting diodes (LEDs); W+UV, white plus UV-A LEDs; W+FR, white plus far-red LEDs; W+FR+UV, a combination of white, UV-A, and far-red LEDs. Lettuce grown under natural sunlight only was used as the control. Different letters on top of the bars denote significant differences at the level of *p* < 0.05 according to the least significant difference test.

The light spectrum had a significant impact on pigment content in greenhouse-grown lettuce (Figure 1). Among all the treatments, the W+FR treatment induced the highest chlorophyll and total chlorophyll contents in lettuce, with no significant differences among the W, W+UV, and W+FR+UV treatments, with chlorophyll contents of 26.8%, 31.7%, and 41.5% higher than in the control, respectively. Chlorophyll b content did not significantly differ among the W+UV, W+FR, and W+FR+UV treatments but was significantly higher under these three treatments than under the W treatment. The total chlorophyll content of greenhouse-grown lettuce under the W+FR treatment was the highest—39.1%, 24.6%, and 16.2% higher than that under the W, W+UV, and W+FR+UV treatments, respectively. The trend of carotenoid content in lettuce leaves was similar to that of chlorophyll content, with significant increases in carotenoid content of leaves under all supplementary treatments. The carotenoid content of lettuce under the W+FR treatment was the highest—50.0%, 50.0%, and 28.6% higher than that under the W, W+UV, and W+FR+UV treatments, respectively. No significant differences were observed in the carotenoid content of lettuce between the W and W+UV treatments.

### 2.3. Effects of Supplementary Light on Biomass and Root Activity of Greenhouse-Grown Lettuce

The biomass of lettuce was higher under supplementary light treatments than under the control treatment (Table 2). In addition, the fresh and dry weights of lettuce were lower under the W treatment compared with other supplementary treatments. The shoot fresh/dry weight and root fresh/dry weight of lettuce were the greatest under the W+FR treatment—4.0, 6.0, 8.0, and 12.4 times higher than the control, respectively. The shoot fresh weight of lettuce under the W+UV and W+FR+UV treatments was significantly higher than that under the W treatment. In addition, the shoot and root dry weights of lettuce showed no significant difference between the W+UV treatment and the W+FR+UV treatment. The root fresh weight of lettuce under the W+UV treatment was increased by 14.5% compared with that under the W+FR+UV treatment.

**Figure 2:**
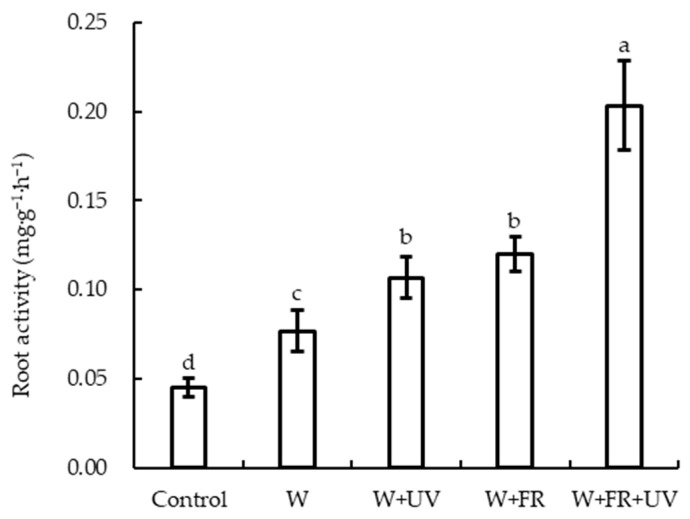
Root activity of greenhouse-grown lettuce under five different treatments. W, white light-emitting diodes (LEDs); W+UV, white plus UV-A LEDs; W+FR, white plus far-red LEDs; W+FR+UV, a combination of white, UV-A, and far-red LEDs. Lettuce grown under natural sunlight only was used as the control. Different letters on top of the bars denote significant differences at the level of *p* < 0.05 according to the least significant difference test.

The root activity of lettuce was higher under the W+FR+UV treatment than under all other treatments, with no significant difference in the root activity of lettuce between the W+UV and W+FR treatments; although, it was significantly higher under these two treatments than that under the W treatment and the control (Figure 2). The root activity of lettuce grown under the W+FR+UV treatment was increased by 81.8% and 66.7% compared with that under the W+UV treatment and the W+FR treatment, respectively. Similarly, the root activity of lettuce grown under the W treatment was 60% higher than that of the control. However, the root activity of lettuce grown under the W treatment was significantly lower than that of lettuce grown under other supplementary treatments.

### 2.4. Impacts of Supplementary Light on Nutritional Quality of Greenhouse-Grown Lettuce

**Figure 3:**
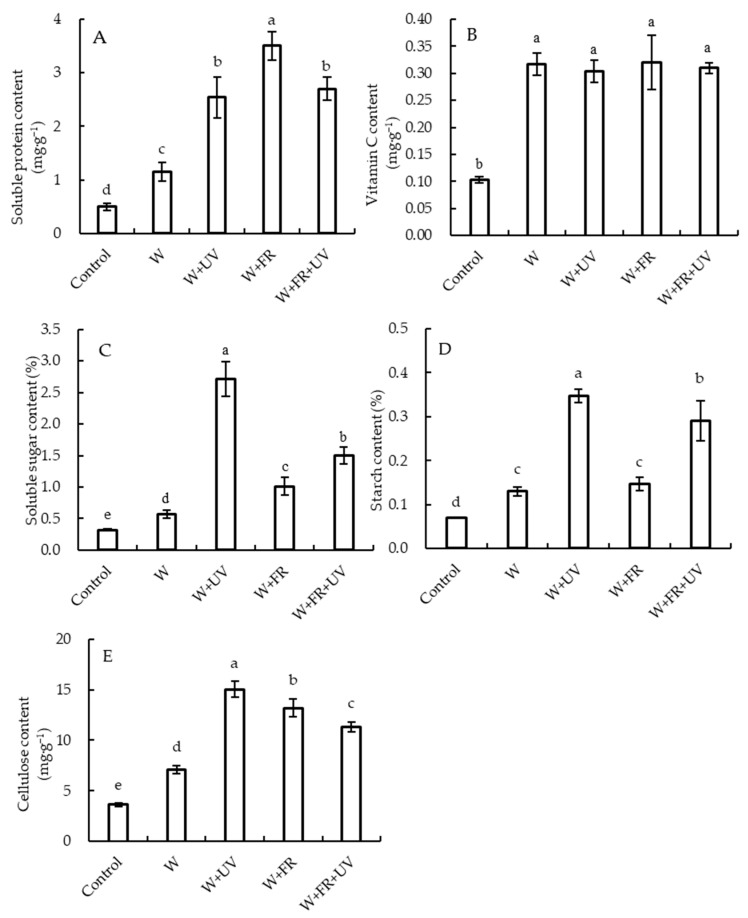
Nutrient contents of greenhouse-grown lettuce under five different treatments. (**A**) Soluble protein content. (**B**) Vitamin C content. (**C**) Soluble sugar content. (**D**) Starch content. (**E**) Cellulose content. W, white light-emitting diodes (LEDs); W+UV, white plus UV-A LEDs; W+FR, white plus far-red LEDs; W+FR+UV, a combination of white, UV-A, and far-red LEDs. Lettuce grown under natural sunlight only was used as the control. Different letters on top of the bars denote significant differences at the level of *p* < 0.05 according to the least significant difference test.

A significant effect of supplementary light treatments on several nutritional qualities of lettuce was observed (Figure 3). The soluble protein content in lettuce grown under the W+FR treatment was the highest—204.3%, 37.8%, and 29.6% higher than that under the W, W+UV, and W+FR+UV treatments, respectively. No significant differences were observed in lettuce vitamin C content among the four supplementary light treatments, but it was significantly higher under the four tested treatments than under the control. In addition, the contents of soluble sugar, soluble starch, and cellulose in lettuce were the highest under the W+UV treatment and the lowest under the W treatment.

### 2.5. Correlation Analysis

**Figure 4:**
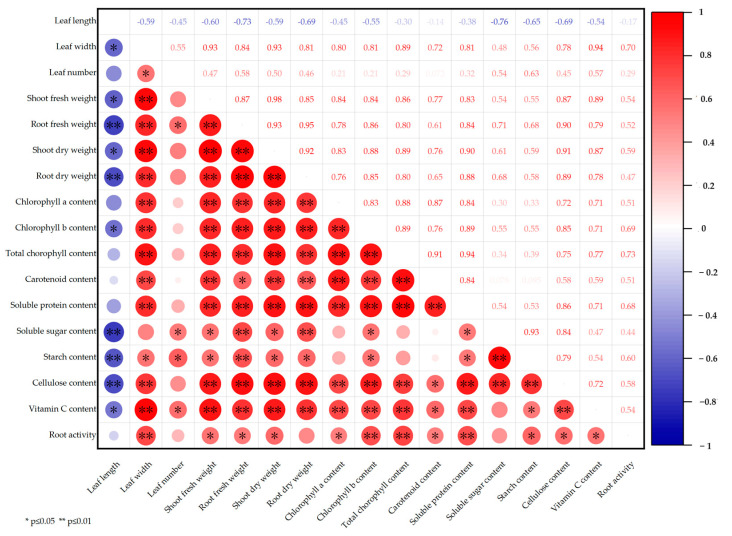
Correlation heat map of 17 growth and nutritional quality indicators of lettuce grown under different supplementary light treatments.

We also performed correlation analysis to examine the correlations among the leaf morphology, biomass, pigment contents, nutrient contents, and root activity of lettuce (Figure 4). Leaf width was significantly positively correlated with shoot fresh weight, shoot dry weight, total chlorophyll content, and vitamin C content, with correlation coefficients of 0.93, 0.93, 0.89, and 0.94, respectively. The root fresh weight was positively correlated with shoot dry weight, root dry weight, and cellulose content but negatively correlated with leaf length. Highly significant positive correlations were observed between biomass and chlorophyll a, chlorophyll b, and total chlorophyll. Carotenoid content was significantly positively correlated with chlorophyll content. Leaf length exhibited a significant negative correlation with soluble sugar content, starch content, and cellulose content, with correlation coefficients of −0.76, −0.65, and −0.69, respectively.

### 2.6. Principal Component Analysis

A principal component analysis (PCA) was performed to evaluate the contributions of each component to differences between the control and supplementary light treatments (Table 3). The contribution rates of the two principal components accounted for 75.85% and 15.13% of the total variance, respectively.

Based on the correlation analysis results, 17 indicators that were highly correlated with the growth and quality of lettuce were selected for subsequent analysis, including the leaf morphological traits, biomass, photosynthetic pigment contents, nutritional contents, and root activity. Further dimensionality reduction analysis of the 17 selected growth and quality indicators of lettuce was performed using SPSS 26.0 software (IBM, Inc., Chicago, IL, USA) to obtain two dimensions (FAC1 and FAC2, corresponding to PC1 and PC2, respectively) (Table 4). The overall growth and quality indicators of lettuce under supplementary light treatments were superior to those under the control treatment. The optimal growth and nutritional quality indicators of lettuce were obtained under the W+FR treatment, with scores of 2.97 for FAC1 and 2.20 for FAC2. The composite score of the W+FR treatment (2.58) ranked first among all supplementary light treatments. The W treatment had a composite score of −0.83, ranking last among all supplementary light treatments.

### 2.7. Heat Map Analysis

**Figure 5:**
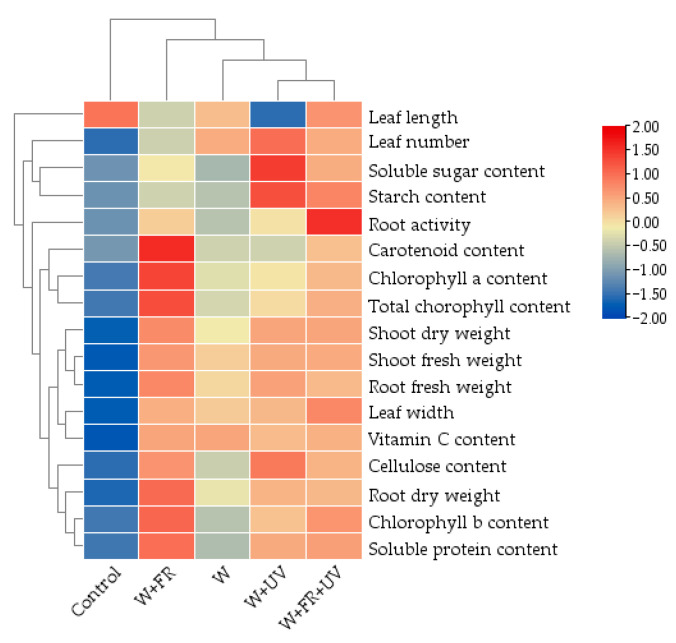
Clustering heat map of 17 growth and nutritional quality indicators of lettuce grown under different supplementary light treatments. W, white light-emitting diodes (LEDs); W+UV, white plus UV-A LEDs; W+FR, white plus far-red LEDs; W+FR+UV, a combination of white, UV-A, and far-red LEDs. Lettuce grown under natural sunlight only was used as the control.

In the clustering heat map, all indicators under all treatments are grouped into four clusters (Figure 5), with the control cluster separated from the supplementary treatments. The W+UV cluster and the W+FR+UV cluster are the closest in terms of the measured parameters. The W+FR treatment cluster is separated from the other clusters due to the high contents of chlorophyll, carotenoid, and soluble protein in greenhouse-grown lettuce under this treatment.

## 3. Discussion

Supplemental lighting in greenhouses during seasons with insufficient light can increase light intensity, regulate light duration, and improve light quality according to crop growth needs [39]. Previous studies have indicated that supplementary lighting leads to shorter plant height and hypocotyl length of vegetable seedlings, resulting in compact morphological features [40]. Our results show that the leaf length of greenhouse-grown lettuce under W treatment was shorter than that of lettuce grown without supplementary light (Table 1), indicating that increasing the daily light integral using supplementary light was beneficial in terms of improving morphological features of plants grown under insufficient light. A similar trend was also observed in lettuce seedlings grown under different daily light integrals [41]. The light spectrum is a critical signal that influences plant growth, development, and metabolic processes [42]. In this study, the leaf length of lettuce under the W+FR treatment was not significantly different from that under the W treatment, but the leaf length under the W+UV treatment was significantly shorter than that under the W treatment. Supplemental light treatment resulted in a significantly larger leaf width than the control, and the leaf width of lettuce treated with W+FR and W+FR+UV was significantly increased compared with that treated with W. These results are consistent with a previous report that far-red light positively influenced leaf width, resulting in a larger leaf area [25]. Far-red light is generally considered to lead to shade-avoidance responses, such as leaf elongation. Far-red light is related to the equilibrium of Pfr and Pr in plants, which can transform into one another by absorbing red light or far-red light, respectively [3]. UV light has been reported to promote plant growth [28]. However, whether far-red light and UV light have a synergistic effect remains unclear. One previous study showed that UVA can activate cryptochrome (a blue light receptor) to mediate the inhibition of plant elongation [16]. Despite sharing a common photoreceptor with blue light, UVA appears to have greater inhibitory effects on plant elongation than blue light, as adding a low level of UVA to blue light can inhibit plant elongation to some degree [43]. Recent studies have indicated that adding FR light to white LED can increase plant height in most microgreen species [27,44], possibly because increased FR light may reduce phytochrome activity, triggering shade-avoidance responses so that plants grow higher to compete for the light source, with elongated stems and petioles [45]. Our results also show that supplementary light significantly increased the leaf width of lettuce, thereby increasing leaf area and eventually improving the light-energy-use efficiency of plants.

The photosynthetic capacity of plants depends on abiotic factors such as light quality and quantity [46]. Chlorophylls and carotenoids are two classes of photosynthetic pigments in higher plants and play vital roles in light absorption of plants; the content and composition of photosynthetic pigment directly affect leaf photosynthetic capacity [33,47]. The photosynthesis rate and biomass production of plants are also largely dependent on the photosynthetic pigment of leaves [48]. The chlorophyll content of lettuce grown under supplementary light was significantly higher compared with the control (Figure 1). Similar trends were observed under supplementary lighting in tomato [49] and cucumber seedlings [50] grown under greenhouse conditions. Light intensity and quality can regulate chlorophyll synthesis through a physiological process mediated by phytochromes [51]. Light quality affects the chlorophyll contents of plants, as supported by our results showing that under the white light supplementation condition, the chlorophyll a, chlorophyll b, and total chlorophyll contents of lettuce significantly increased compared with those in the sunlight group, and these three indicators of lettuce were the highest under the W+FR treatment (Figure 2). The present study shows that the supplementation of far-red light at adequate PPFD can increase the chlorophyll content of plants. One previous study also showed that the temporary addition of FR light significantly stimulates leaf instantaneous photosynthetic rates under different supplementary light durations [38]. This result is in agreement with another previous report that FR light is required for efficient photochemistry and photosynthesis because FR light preferentially excites photosystem I [31]. In addition, the chlorophyll a, chlorophyll b, and total chlorophyll contents of lettuce under the W+UV and W+FR+UV treatments were significantly higher than those under the control treatment but lower than those under W+FR, indicating that the impact of UV on plant chlorophyll contents was weaker than that of FR. Our findings indicate that the carotenoid content of lettuce was higher in all supplemental light treatments than under natural light. A previous study indicated that plants acclimated to high levels of light generally have higher carotenoid contents than those of low-light-acclimated plants [52], which is consistent with our results. In addition, the carotenoid content of lettuce under the W+FR treatment was the highest among all treatments. Some studies have shown that adding a certain percentage of far-red light can increase the carotenoids of potato leaves [53] and red leaf lettuce [30]. However, Chen et al. [54] indicated that the carotenoid content of lettuce (‘Green Oak Leaf’) under the W+FR treatment was lower than that of lettuce grown under the W treatment, indicating that the responses of pigment contents to the light spectrum in lettuce may be dependent on the variety/genotype or the basal light.

A suitable daily light integral can improve plant growth, shorten seedling cultivation time, and increase light-energy-use efficiency [55,56]. The fresh and dry weights of plant shoots have been reported to increase significantly with an increase in light intensity [40]. In this study, supplementary light treatments significantly increased the biomass of lettuce compared to the control, and the addition of UVA or FR to white light increased the biomass of lettuce compared with treatment with white light alone. UVA has been reported to increase fresh and dry weights and promote biomass accumulation in lettuce [57]. The addition of FR light can increase the shoot dry weight of geraniums and snapdragons, which can be attributed to the fact that a larger leaf area increases the light capture efficiency [36]. Far-red light treatment can also increase fresh and dry weights, leaf area, shoot height, internodal length, bioactive compound accumulation, and mitotic cell division in lettuce [58,59,60]. In the present study, adding FR light to white light also increased the plant biomass of the greenhouse-grown lettuce, with the largest increment among all light treatments. Zhen and van Iersel [31] observed that the net photosynthetic rate and photochemical efficiency of PSII of lettuce increased when far-red light was added to white LED light, indicating that far-red photons effectively induce phytochrome-mediated responses, promote photosynthesis, and increase biomass production [2,37]. The increase in the photochemical efficiency of PSII after adding far-red light is thought to be caused by preferential excitation of PSI by far-red light [61]. Moreover, the dry and fresh weights of the shoot and root of lettuce under W+FR+UV were significantly lower than those under W+FR. This result indicates that there was no interaction between FR and UVA when they were simultaneously combined with other light; similar results were reported in a recent study [36]. Light quality has an important influence on the growth and development of plant roots [62]. The root activity of cucumber seedlings exhibited a trend of first increasing and then decreasing with the increasing blue light [51]. Our data show that the root activity of greenhouse-grown lettuce was significantly higher under the W+FR+UV treatment than under other treatments, indicating that UV and FR had positive effects on the root activity of the plant.

Light affects the synthesis and distribution of primary and secondary metabolites in plants. Our data show that the contents of various nutrients in lettuce increased significantly under all supplementary light treatments. The highest soluble sugar, starch, and cellulose contents in lettuce plants were observed under the W+UV treatment, and the highest soluble protein content was observed under the W+FR treatment (Figure 3). Our findings are consistent with some previous reports that supplementing sunlight with UV significantly increases protein accumulation in some plants [30,63]. In this study, the soluble sugar content in lettuce under the W+UV treatment was significantly higher than that under the W+FR treatment, suggesting that UV addition had a stronger positive effect on soluble sugar content than FR addition. UV supplementation has been reported to increase soluble sugar contents [25]. The glucose and fructose contents in tomato fruits are negatively correlated with FR light intensity [64]. Previous studies have shown that supplemental FR light increases the starch content of basil leaves, protecting the structural and functional integrity of photosynthetic cells and potentially improving their stress tolerance [65,66]. Our data show that the vitamin C content in lettuce was significantly higher under the supplementary light treatments than under the control treatment, with the highest vitamin C observed under the W+FR treatment (Figure 3). Vitamin C biosynthesis is more efficient under far-red light, and FR light can enhance the activities of some enzymes through a series of signal reactions [55].

Supplementing UV and FR light affects multiple morphological parameters and nutritional quality traits of lettuce (Figure 4 and Figure 5). The correlation heat map shows that the contents of cellulose, vitamin C, and soluble protein are significantly correlated with biomass and chlorophyll content. The clustering heat map shows that far-red light has a positive effect on the chlorophyll content, biomass, and soluble protein of lettuce. UV light promotes the soluble sugar and starch contents of lettuce. The combination of white, far-red, and UV lights dramatically increases plant root activity.

## 4. Materials and Methods

### 4.1. Plant Materials

Lettuce (*Lactuca sativa* L. cv. Dasusheng) seeds were grown in 72-cell plug trays filled with mixed peat (The Pindstrup Group, Kongersle, Denmark), vermiculite (Shandong Lige Technology Co., Ltd., Jinan, China), and perlite (Shandong Lige Technology Co., Ltd., Jinan, China) (3:1:1, *v*/*v*/*v*) in a walk-in growth chamber (Qingdao Agricultural University, Qingdao, China). A light intensity of 200 μmol m^−2^ s^−1^ with a 16 h d^−1^ photoperiod provided by white LEDs (Weifang Hengxin Electric Appliance Co., Ltd., Weifang, China) was employed for the seedling stage. Then, lettuce seedlings with three true leaves were transplanted into 2.2 L plastic pots (diameter, 17.5-cm; depth, 14.5-cm) filled with mixed peat, vermiculite, and perlite (3:1:1, *v*/*v*/*v*) (one day before treatment). The plastic pots were kept in a Venlo-type greenhouse at Qingdao Agricultural University (36°19′ N, 120°23′ E), Qingdao, Shandong Province, China, at a temperature of 22 ± 3 °C during the day and 18 ± 3 °C at night, and the relative humidity was maintained at 60–70% for 25 days. The gutter height, ridge height, and floor area of the glass greenhouse were 4.5 m, 5.3 m, and 2736 m^2^, respectively. Lettuce was cultivated using Hoagland’s nutrient solution according to a previously reported method [12].

### 4.2. Treatment Design

**Figure 6:**
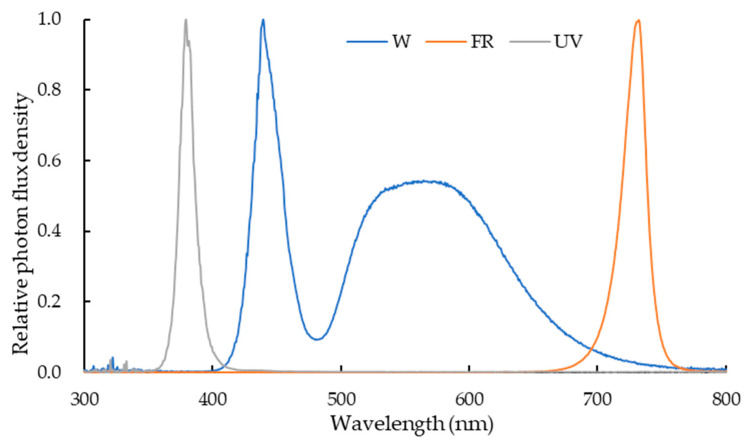
Spectral distribution of supplementary white light-emitting diodes (LEDs) with a peak wavelength of 440 nm, far-red LEDs (FR) with a peak wavelength of 730 nm, and UV-A LEDs (UV) with a peak wavelength of 380 nm.

The average daily light integral (DLI) of solar light inside the greenhouse was 5.0 mol m^−2^ d^−1^ during the experimental period (15 November to 9 December 2022). In this study, supplemental DLI was set as 6.52 mol m^−2^ d^−1^, light intensity was set as 151 µmol m^−2^ s^−1^, and the photoperiod was set as 12 h d^−1^ (04:00–10:00 and 14:00–20:00) according to a previous report [67]. The four following light supplementation treatments were applied: white LEDs (W) (Weifang Hengxin Electric Appliance Co., Ltd., Weifang City, China); white plus far-red LEDs (W+FR) (Xiamen Lumigro Technology Co., Ltd., Xiamen City, China); white plus UV-A LEDs (W+UVA) (Xiamen Lumigro Technology Co., Ltd., Xiamen City, China); and the combination of white, far-red, and UV-A LEDs (W+FR+UVA). The light intensity of UVA and far-red light was 10 and 50 µmol m^−2^ s^−1^, respectively. Additionally, lettuce grown without supplementary light was used as the control (DLI at 5.0 mol m^−2^ d^−1^ from sunlight only for 25 days). The spectral distribution of the LEDs (Figure 6) applied in the present study as supplementary light was measured with a spectrometer (AvaSpec-ULS2048-USB2, Avantes Inc., the Netherlands) above the plant canopy. The peak wavelengths of the white, UVA, and far-red LEDs were 440 nm, 380 nm, and 730 nm, respectively. The experiments were conducted with 3 replicates per treatment and 15 lettuce plants per replicate.

### 4.3. Growth Indicator Measurement

#### 4.3.1. Plant Morphology and Growth Characteristics

Leaf length and leaf width of the maximum leaf blade and leaf and root dry and fresh weights of lettuce were measured at harvest (25 days after treatment) using an electronic analytical balance (JY20002, Shanghai Hengping Instrument Co., Ltd., Shanghai, China). The fresh leaves and roots were dried in an oven at 105 °C for 3 h, then dried at 80 °C for 72 h to measure their dry weights.

#### 4.3.2. Pigment Contents

The chlorophyll a, chlorophyll b, and carotenoid contents of lettuce were determined spectrophotometrically according to the method reported by Lichtenthaler and Wellburn [68].

#### 4.3.3. Root Activity

The root activity of lettuce was determined using the triphenyl tetrazolium chloride (TTC) method as described by Li [69] and Yan et al. [5]. The absorbance of the test samples was measured at 485 nm at room temperature using a spectrophotometer (UV1810, Shanghai Yoke Instrument Co., Ltd., Shanghai, China).

#### 4.3.4. Starch, Cellulose, Vitamin C, Soluble Sugar, and Soluble Protein

The starch content of the lettuce leaf samples was measured according to a previously reported method [70]. The Updegraff method was applied to determine the cellulose content of lettuce [71]. Vitamin C, soluble sugar, and soluble protein contents were determined by the 2, 6-dichlorophenol indophenol titration method, anthrone sulfuric acid colorimetry method, and Coomassie brilliant blue G-250 dye method, respectively, according to Li [69] and Zhang et al. [72]. Lettuce leaves were cut into small pieces and mixed for the measurements. Absorbances at wavelengths of 630 nm and 595 nm were used to measure soluble sugar and soluble protein contents of lettuce leaves with a spectrophotometer (UV1810, Shanghai Yoke Instrument Co., Ltd., Shanghai, China).

### 4.4. Statistical Analysis

Data are expressed as the means ± standard deviations (SD) of three replicates. One-way analysis of variance (ANOVA) and the least significant difference (LSD) test (*p* < 0.05) were carried out using SPSS 26.0 software (IBM, Inc., Chicago, IL, USA) to reveal differences among groups. The correlation analysis heat map and the clustering heat map were plotted using Origin 2023 (Origin Lab Corporation, Northampton, MA, USA) software and TBtools (http://cj-chen.github.io/TBtools/, accessed on 10 July 2023). Euclidean distance was used, and the complete clustering method was applied as reported by Gao et al. [73].

## 5. Conclusions

Overall, this study reveals that the W+FR treatment improved pigment content, biomass, and soluble protein content in greenhouse-grown lettuce and that the soluble sugar, starch, and cellulose contents were higher in lettuce grown under the W+UV treatment than that grown under the W+FR treatment. Under the W+FR+UV treatment, the root activity of greenhouse-grown lettuce was the highest. Our results indicate that the addition of far-red light to white light improved the growth performance of greenhouse lettuce and that the addition of UV-A light also had positive effects on the nutritional qualities of lettuce. Our findings provide support for the establishment of guidelines for the application of light-supplementation strategies in greenhouse lettuce production.

## Figures and Tables

**Table 1:** Leaf morphological traits of greenhouse-grown lettuce under four supplementary light treatments.

Treatment	Leaf Length (cm)		Leaf Width (cm)		Leaf Number	
Control	19.6 ± 1.0	a	9.0 ± 0.6	c	5.0 ± 1.0	b
W	17.4 ± 0.7	b	14.6 ± 0.3	b	6.3 ± 0.6	a
W+UV	14.7 ± 1.8	c	14.7 ± 0.2	b	6.7 ± 0.6	a
W+FR	17.4 ± 1.1	b	16.1 ± 0.5	a	5.7 ± 0.6	ab
W+FR+UV	17.8 ± 1.0	ab	16.2 ± 0.5	a	6.3 ± 0.6	a

Notes: W, white light-emitting diodes (LEDs); W+UV, white plus UV-A LEDs; W+FR, white plus far-red LEDs; W+FR+UV, a combination of white, UV-A, and far-red LEDs. Lettuce grown under natural sunlight only was used as the control. Different lowercase letters within one column indicate significant differences at the level of *p* < 0.05 according to the least significant difference test.

**Table 2:** Biomass of greenhouse-grown lettuce under four supplementary light treatments.

Treatment	Shoot Fresh Weight (g/Plant)		Root Fresh Weight (g/Plant)		Shoot Dry Weight (g/Plant)		Root Dry Weight (g/Plant)	
Control	9.40 ± 0.50	d	0.67 ± 0.12	e	0.64 ± 0.02	d	0.05 ± 0.01	d
W	34.53 ± 0.38	c	3.68 ± 0.10	d	2.61 ± 0.05	c	0.35 ± 0.01	c
W+UV	42.35 ± 0.43	b	5.18 ± 0.23	b	3.90 ± 0.06	b	0.49 ± 0.06	b
W+FR	47.33 ± 0.90	a	6.02 ± 0.33	a	4.50 ± 0.13	a	0.67 ± 0.01	a
W+FR+UV	42.63 ± 0.68	b	4.43 ± 0.16	c	3.90 ± 0.02	b	0.48 ± 0.02	b

Notes: W, white light-emitting diodes (LEDs); W+UV, white plus UV-A LEDs; W+FR, white plus far-red LEDs; W+FR+UV, a combination of white, UV-A, and far-red LEDs. Lettuce grown under natural sunlight only was used as the control. Different lowercase letters within one column indicate significant differences at the level of *p* < 0.05 according to the least significant difference test.

**Table 3:** Principal component analysis of greenhouse-grown lettuce.

Principal Component	Eigenvalue	Contribution Rate	Cumulative Contribution Rate
		(%)	(%)
1	12.90	75.85	75.85
2	2.57	15.13	90.98

**Table 4:** Factor analysis and composite scores of four supplementary light treatments.

Treatment	FAC1	FAC2	Composite Score
Control	−5.82	0.31	−4.37
W	−1.07	−0.12	−0.83
W+UV	2.05	−2.31	1.21
W+FR	2.97	2.20	2.58
W+FR+UV	1.87	−0.08	1.41

Notes: FAC1 and FAC2 represent factor scores of two principal components. W, white light-emitting diodes (LEDs); W+UV, white plus UV-A LEDs; W+FR, white plus far-red LEDs; W+FR+UV, a combination of white, UV-A, and far-red LEDs. Lettuce grown under natural sunlight only was used as the control.

## Data Availability

Please contact the corresponding author for any additional information.
